# Exploring the gut microbiota’s effect on temporomandibular joint disorder: a two−sample Mendelian randomization analysis

**DOI:** 10.3389/fcimb.2024.1361373

**Published:** 2024-08-12

**Authors:** Kai Zhao, Shuaiqi JI, Han Jiang, Yunzhu Qian, Weibing Zhang

**Affiliations:** ^1^ Department of Stomatology, The Fourth Affiliated Hospital of Soochow University, Suzhou Dushu Lake Hospital, Medical Center of Soochow University, Suzhou, China; ^2^ Fujian Key Laboratory of Oral Diseases and Stomatological Key lab of Fujian College and University, School and Hospital of Stomatology, Fujian Medical University, Fuzhou, China

**Keywords:** gut microbiota, temporomandibular joint disorder, Mendelian randomization, causal inference, genetic variation

## Abstract

**Background:**

Temporomandibular joint disorders (TMD) are highly prevalent among people. Numerous investigations have revealed the impact of gut microbiota in many diseases. However, the causal relationship between Temporomandibular joint disorders and gut microbiota remains unclear.

**Methods:**

Genome-Wide Association Studies (GWAS) refer to the identification of sequence variations, namely single nucleotide polymorphisms (SNPs), existing across the entire human genome. GWAS data were collected on gut microbiota and TMD. Then, instrumental variables were screened through F-values and removal of linkage disequilibrium. These SNPs underwent mendelian analysis using five mathematical models. Sensitivity analysis was conducted to further verify the stability of the results. Pathogenic factors of TMD mediate the causal relationship between gut microbiota and TMD were explored through a two-step Mendelian randomization analysis. Finally, reverse mendelian analysis was conducted to account for potential reverse effects.

**Results:**

The analysis of the data in this article suggests that some gut microbiota, including Coprobacter, Ruminococcus torques group, Catenibacterium, Lachnospiraceae, Turicibacter, Victivallis, MollicutesRF9, Methanobacteriales, Methanobacteriaceae, FamilyXI, Methanobacteria were identified as risk factors, while Peptococcaceae provides protection for TMD.

**Conclusion:**

The research reveals the relation of gut microbiota in TMD. These findings provide insights into the underlying mechanisms and suggest potential therapeutic strategy.

## 1 Introduction

Temporomandibular joint disorders (TMD) are a collective term for a group of conditions affecting the temporomandibular joint and/or surrounding muscles, characterized by pain and dysfunction (Scrivani et al., 2008; Andre et al., 2022; Thomas et al., 2023). TMD are highly prevalent among people, with children and adolescents also being susceptible to the condition (Kopp et al., 2023). Approximately 5% to 12% of Americans are affected by TMD, with annual total costs for treating TMD reaching around $4 billion (Schiffman et al., 2014; Manrriquez et al., 2021). Women have a higher risk of developing TMD, with prevalence rates ranging from 25% to 40% in population (Murphy et al., 2013; Calixtre et al., 2014; Valesan et al., 2021). Nearly half of individuals diagnosed with TMD experience persistent or recurring symptoms, often leading to a diminished quality of life (List and Axelsson, 2010; Maixner et al., 2011). Treating TMD poses many challenges. Comorbidities are highly prevalent among TMD patients, including headaches, widespread pain, fibromyalgia, neck and back pain, and psychosocial disorders like stress, anxiety, depression (Aggarwal et al., 2006). Prospective and risk assessment studies on oral and facial pain support the biological psychological social model of TMD, emphasizing the role of comprehensive treatment (Fillingim et al., 2018). Traditionally, treatment methods for TMD include cognitive–behavioral therapy, anti-inflammatory drug therapy, splint therapy, minimally invasive, arthroscopic, or open surgery (Manrriquez et al., 2021; Valesan et al., 2021). Despite the availability of numerous treatments for TMD, they often fail to prevent the occurrence or recurrence of the condition. The etiological factors of TMD are still unclear.

The human gut harbors thousands of microbial species, forming a complex ecological community, called the gut microbiome (Lagier and Raoult, 2016). These microorganisms are major mediators of body homeostasis, influencing various physiological activities such as metabolism, barrier homeostasis, inflammation, and hematopoiesis through intestinal and parenteral actions. Recently, the gut microbiota has recently been classified as a “vital organ” due to its establishment of multidirectional and communicative links or axes with other organs through neural, endocrine, humoral, immune, and metabolic pathways. Changes in the microbiome can lead to gut-related problems, but also affects other organ-related diseases, although the actual mechanisms of gut-organ interaction are not fully understood. A recent study had revealed a correlation between the occurrence of TMD and the deregulation of microbial metabolites (Ma et al., 2020). Extensive research on the brain-gut axis underscores the pivotal role of gut microbiota in adjusting the secretion of inflammatory neurotransmitters, which could lead to the release of diverse proinflammatory mediators in the process of TMD (Shrivastava et al., 2021; Aburto and Cryan, 2024).

Widespread debate still exists regarding whether there is a causal relationship between abnormal microbial communities associated with diseases. The substitution of a nucleotide in the genome can significantly alter the function of an organism. Single nucleotide polymorphisms (SNPs) are commonly present in microorganisms and can endow bacteria with antibiotic resistance or the ability to infect new host species. The diversity of SNPs may reflect the correlation between host microbial interactions. Current research lacks comprehensive evidence to confirm between gut bacteria and TMD. Mendelian randomization (MR) is a crucial instrument for exploring the potential causal relationship between exposure and outcomes using instrumental variables (IV) (Davey and Hemani, 2014; Swerdlow et al., 2016; Bowden and Holmes, 2019). This approach is based on the fair and random allocation of alleles to the next generation, and has emerged as a powerful tool for exploring the intricate connections between complex traits and diseases (Bowden and Holmes, 2019). MR is currently widely used in oncology, neuroscience, cardiovascular and genetics. Its advantage in exploring causal relationships makes it have broad prospects (Swerdlow et al., 2016; Bowden and Holmes, 2019; Ouyang et al., 2022). With the continuous advancement of technology and the ongoing refinement of research methods, such as Genome-wide association studies, MR becomes an important method in the biomedical fields. In this research, we aimed to explore the causality between the intestinal microbiome and TMD by Mendelian randomization.

## 2 Methods

### 2.1 Data sources

A summary of the GWAS data was originated from MiBioGen (https://mibiogen.gcc.rug.nl/), which comprised 16S rRNA sequencing and genotyped their participants with full-genome SNP arrays. The sequencing of gut microbiota in the Mibiogen database offered a comprehensive catalog of gene variations associated with gut microbiota (Kurilshikov et al., 2021). These variations were derived from 18,340 participants representing 24 countries, individuals from Asian, American, and African populations. Currently, this database is recognized as the most extensive and comprehensive among comparable database. The database comprises an analysis of the variable regions V3, V4-V1, and V2-V16 of the 16S rRNA gene from microorganisms. The results are categorized into five levels: genus, family, order, class, and phyla, encompassing a total of 211 species. Our outcomes were sourced from the IEU Open GWAS database (https://gwas.mrcieu.ac.uk/datasets), a comprehensive database (Zheng et al., 2022b). This dataset includes a European population with a comprehensive sample size of 134,280 individuals, including 2,730 diagnosed with TMD and 131,550 control individuals, providing a total of 16,379,953 SNPs. All data in this study were derived from public publications, thus ethical approval or patient consent is not required for analysis.

### 2.2 Experimental design

**Figure 1:**
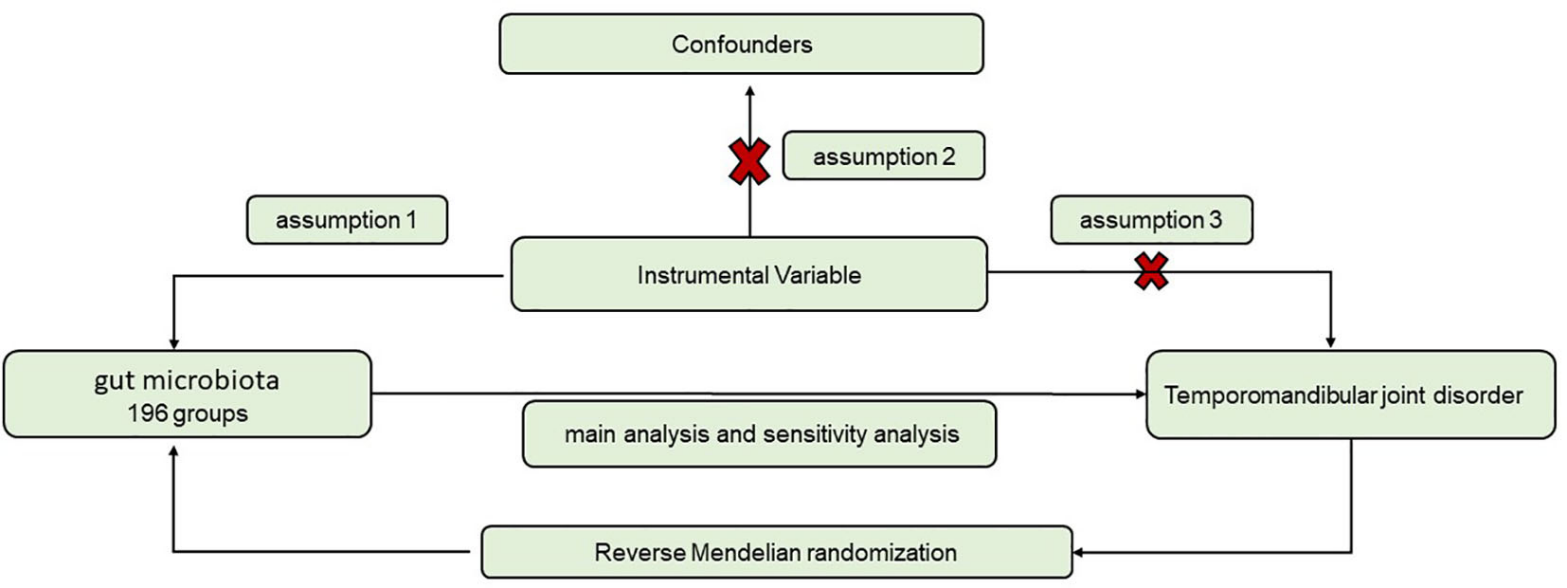
Flowchart for the study of the association between gut microbiota and TMD. (1) SNPs are closely related to the exposure; (2) SNPs are unrelated to the outcome; (3) SNPs are unrelated to confounding factors.

GWAS refer to the identification of sequence variations, namely SNPs, existing across the entire human genome. In order to investigate the causal connection between gut microbiota and TMD, we conducted a bidirectional dual-sample MR study utilizing this GWAS dataset (**Figure 1**). Individuals were grouped into different genotype groups based on SNPs strongly associated with TMD. By simulating random distribution of genetic information from parental genomes to offspring, occurrence rates of TMD among different genotype groups were compared. The IVs need to fit three main assumptions (1): SNPs are closely related to the exposure (2); SNPs are unrelated to the outcome (3); SNPs are unrelated to confounding factors. For exposure SNPs, the pooling process is conducted according to SNP loci with P < 1 × 10-5, while ensuring R2 < 0.001 and genetic distance of 10000 to eliminate linkage disequilibrium (LD). After eliminating the SNPs of echo sequence, the remaining SNPs were chosen. This step ensured that the IVs fits assumption. Weak instrumental variables are genetic variations with lower explanatory power for exposure. They are associated with exposure, but the strength of this association is limited (Davey and Hemani, 2014). According to literature reports, F > 10 was considered to be the standard for excluding weak instrumental variable bias (Burgess and Thompson, 2011; Zheng et al., 2022a). Next, the screened SNPs were used as IVs to evaluate the relationship between gut microbiota and TMD.

### 2.3 Statistical analysis

In this investigation, diverse statistical approaches were utilized to assess the causal link between gut microbiota and TMD. These methods encompassed inverse variance weighted (IVW), simple mode, MR- Egger regression, weighted median (WM), and weighted model (WME) (Hemani et al., 2018). The characteristic of IVW method is that it does not consider the presence of intercept terms during regression, and it uses the inverse of the outcome variance (the square of the standard error) as weights for fitting. IVW was seemed as the primary method, and the other methods as **Supplementary Methods** to evaluate the relationship between exposure and outcomes under various conditions (Carter et al., 2021). If P < 0.05, the outcome was deemed statistically significant and expressed in terms of odds ratio (OR) along with the corresponding 95% confidence interval (95% CI).

### 2.4 Sensitivity analysis

Sensitivity analysis was performed to validate the stability of this findings and to assess potential biases and heterogeneity in each IVs. Egger regression analysis is a type of weighted linear regression where the standard normal deviation of the effect value serves as the dependent variable, while its accuracy serves as the independent variable. The MR-Egger method was employed to assess the presence of horizontal pleiotropy. Simultaneously, the Cochran Q test was applied to investigate potential heterogeneity, with significance set at P<0.05. And leave-one-out analysis was conducted, systematically removing each exposure to evaluate results stability. Additionally, the MR-PRESSO test was used to identify outliers and recompute the results. The analyses were performed by R version 4.2.3, with the “TwoSampleMR” and “MRPRESSO” software packages (Burgess and Thompson, 2015).

### 2.5 Two-step mendelian randomization

The overall impact of exposure on the outcome can be decomposed into direct effects and synergistic effects. In order to detect the synergistic effect on TMD, two-step MR was used to examine the mediating effects of other factors (Carter et al., 2021; Zhao et al., 2022). Dentofacial anomalies was considered as TMD influencing factors, serving as an intermediary factor of intestinal flora affecting TMD. Maxillofacial deformities underwent univariate Mendelian randomization, and significant intestinal flora results were tested using the coefficient product method.

### 2.6 Reverse Mendelian randomization

Reverse Mendelian randomization also was conducted to assess the stability of results.

## 3 Results

### 3.1 The screen of IVs

15 groups of unknown data from the gut microbiota were excluded, while 196 data were entered statistical analysis. By computing P values, LD, and conducting F-value tests, we conducted screening on multiple SNPs within 196 genotypes. The SNPs that passed the screening were considered to eliminate instrumental bias and F>10 (**Supplementary Table 1**). All SNPs which were screened would seem as instrumental variables for the subsequent experiments.

### 3.2 Effect of IV in TMD

**Figure 2:**
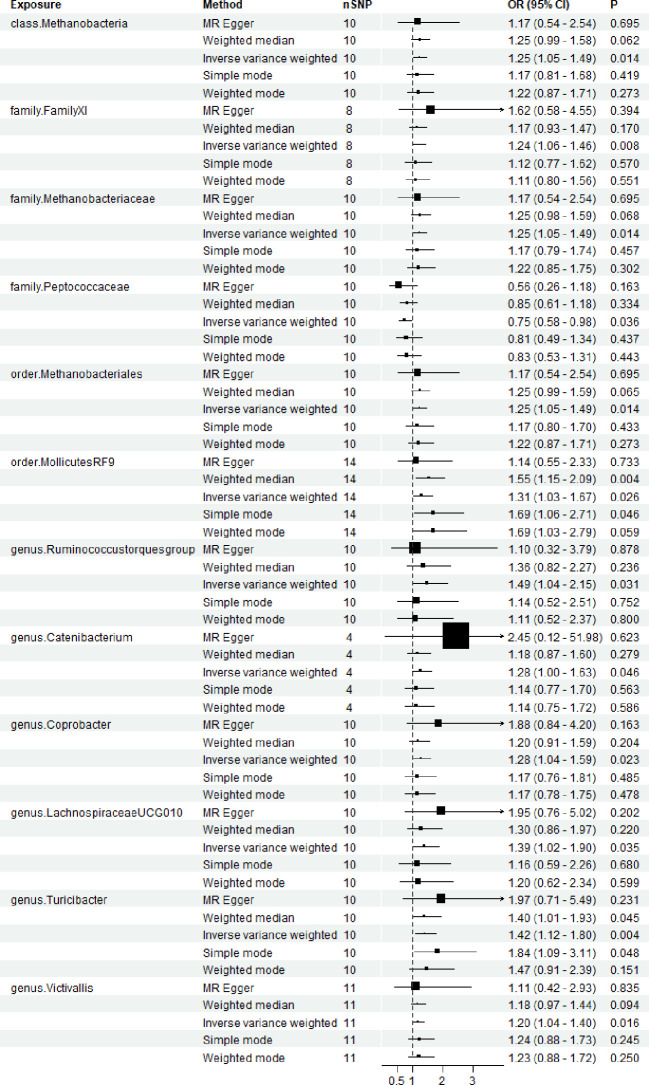
Forest plots for the association of gut microbiota and Temporomandibular joint disorder. OR, odds ratio; CI, confidence interval. P < 0.05.

2601 SNPs were screened from 196 microbial samples. 11 microbial group that showed significant effect on the occurrence of TMD, and the IVW outcomes consistently demonstrated significant effects. The IVW results indicated that the following microbial groups were associated with an increased risk of TMD: genus *Coprobacter* (OR = 1.28, 95% CI (1.04-1.59), P = 0.035), genus *Ruminococcus torques group* (OR = 1.49, 95% CI (1.04-2.15), P = 0.031), genus *Catenibacterium* (OR = 1.28, 95% CI (1.04-1.59), P = 0.023), genus *LachnospiraceaeUCG010* (OR = 1.39, 95% CI (1.02-1.90), P = 0.035), genus *Turicibacter* (OR = 1.42, 95% CI (1.12-1.80), P = 0.004), genus *Victivallis* (OR = 1.20, 95% CI (1.02-1.40), P = 0.016), order *MollicutesRF9* (OR = 1.31, 95% CI (1.31-1.67), P = 0.026), order *Methanobacteriales* (OR = 1.25, 95% CI (1.05-1.49), P = 0.014), family *Methanobacteriaceae* (OR = 1.25, 95% CI (1.05-1.49), P = 0.014), family *FamilyXI* (OR = 1.24, 95% CI (1.06-1.46), P = 0.008), class *Methanobacteria* (OR = 1.25, 95% CI (1.05-1.49), P = 0.014). And one microbial group was demonstrated a significant effect prevent the progression of TMD: family *Peptococcaceae* (OR = 0.75, 95% CI (0.58-0.98), P = 0.036). (**Figure 2**; **Supplementary Table 2**).

### 3.3 Sensitivity analyses

**Figure 3:**
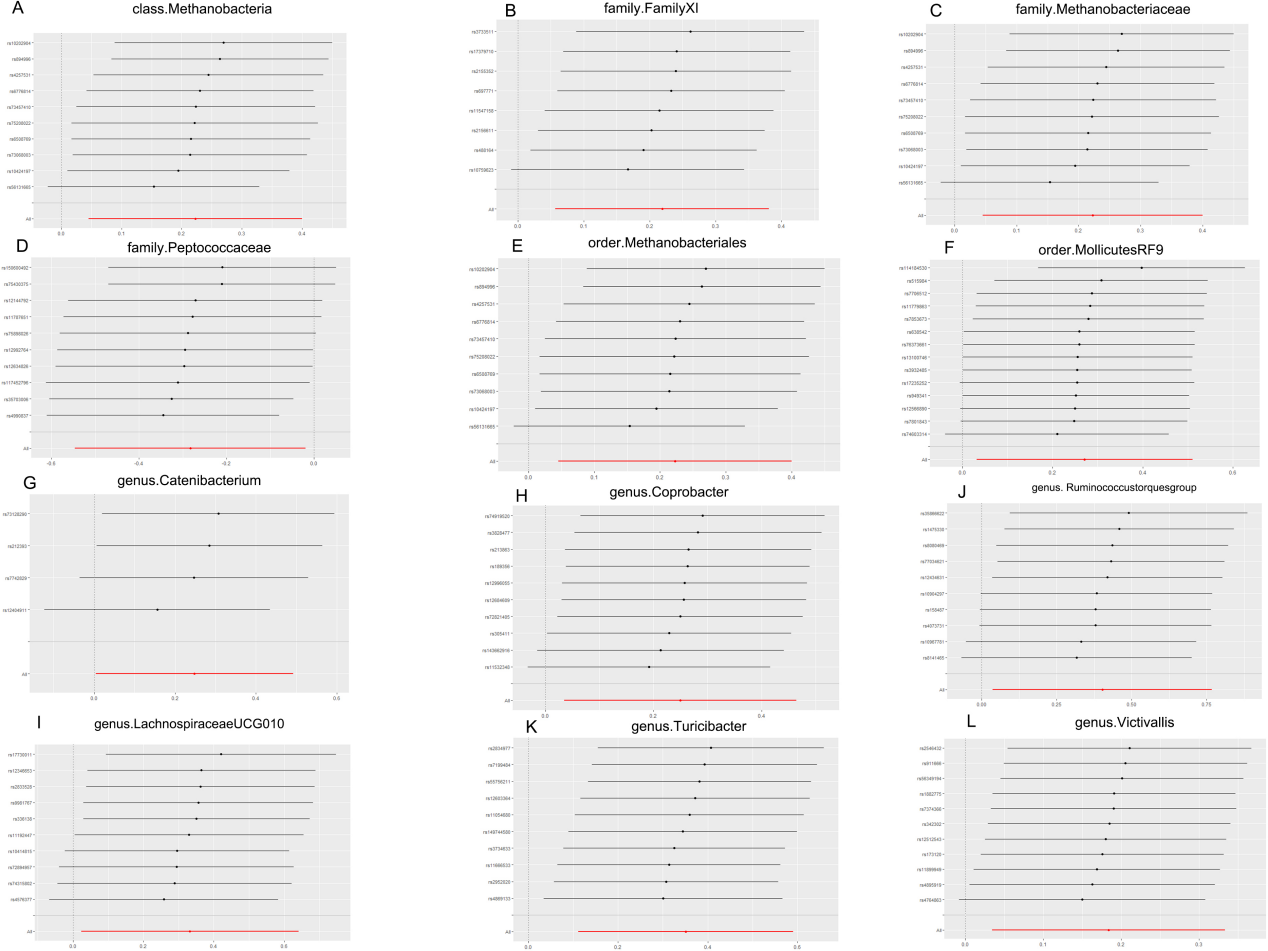
MR leave-one-out sensitivity analysis. **(A)** Leave-one-out sensitivity analysis of the effect of class. Methanobacteria on TMD; **(B)** Leave-one-out sensitivity analysis of the effect of family. FamilyXI on TMD; **(C)** Leave-one-out sensitivity analysis of the effect of family. Methanobacteriaceae on TMD; **(D)** Leave-one-out sensitivity analysis of the effect of family. Peptococcaceae on TMD; **(E)** Leave-one-out sensitivity analysis of the effect of order. Methanobacteriales on TMD; **(F)** Leave-one-out sensitivity analysis of the effect of order.MollicutesRF9 on TMD; **(G)** Leave-one-out sensitivity analysis of the effect of genus. Catenibacterium on TMD; **(H)** Leave-one-out sensitivity analysis of the effect of genus. Coprobacter on TMD; **(I)** Leave-one-out sensitivity analysis of the effect of genus.Lachnospiraceaeucg010 on TMD; **(J)** Leave-one-out sensitivity analysis of the effect of genus. Ruminococcus torques group on TMD; **(K)** Leave-one-out sensitivity analysis of the effect of genus. Turicibacter on TMD; **(L)** Leave-one-out sensitivity analysis of the effect of genus. Victivallis on TMD. TMD, Temporomandibular joint disorder; MR, Mendelian randomization.

**Figure 4:**
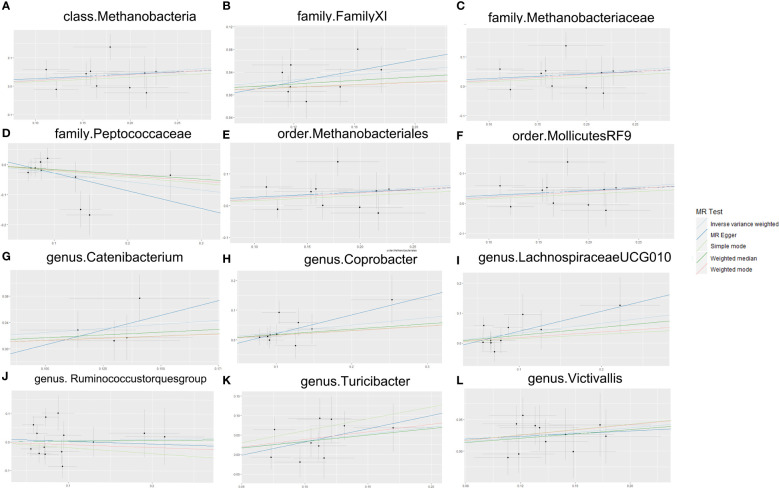
MR Egger sensitivity analysis. **(A)** Egger sensitivity analysis of the effect of class.Methanobacteria on TMD; **(B)** Egger sensitivity analysis of the effect of family.FamilyXI on TMD; **(C)** Egger sensitivity analysis of the effect of family. Methanobacteriaceae on TMD; **(D)** Egger sensitivity analysis of the effect of family. Peptococcaceae on TMD; **(E)** Egger sensitivity analysis of the effect of order. Methanobacteriales on TMD; **(F)** Egger sensitivity analysis of the effect of order.MollicutesRF9 on TMD; **(G)** Egger sensitivity analysis of the effect of genus. Catenibacterium on TMD; **(H)** Egger sensitivity analysis of the effect of genus. Coprobacter on TMD; **(I)** Egger sensitivity analysis of the effect of genus.Lachnospiraceaeucg010 on TMD; **(J)** Egger sensitivity analysis of the effect of genus. Ruminococcus torques group on TMD; **(K)** Egger sensitivity analysis of the effect of genus. Turicibacter on TMD; **(L)** Egger sensitivity analysis of the effect of genus. Victivallis on TMD. TMD, Temporomandibular joint disorder; MR, Mendelian randomization.

According to the results of the Cochran Q test, including both the IVW and Egger methods, P values were not significant. Therefore, the results seemed to be stable, indicating no apparent heterogeneity. Additionally, tests for Leave-one-out demonstrated stable conclusions (**Figure 3**). MR-Egger regression testing further confirmed the stability of the results (**Figure 4**; **Supplementary Table 3**).

### 3.4 Two-step mendelian randomization

Malocclusion exhibited significant relationships with TMD (**Supplementary Figure 3**). The results suggest that that the causal relationship between the three bacteria (*Turicibacter*, *Victivallis*, *Peptococcaceae*) (**Supplementary Figure 4**) and TMD may be partly mediated by maxillofacial deformities.

### 3.5 Reverse MR analysis

In order to test potential reverse causality influencing the previous results, reverse MR analysis was performed to treating significant gut microbiota as the outcome and TMD as the exposure variables. Notably, the analysis of the data did not yield any evidence supporting a reverse causal association between TMD and the identified gut microbiome (**Supplementary Figure 5**).

## 4 Discussion

According to recent epidemiological, cellular biology, and genomics research, a significant portion of the external influences on the human body appears to mediated by the gut microbiota. The gut microbiota, being the largest microbial community, exerts a substantial influence on training host immunity, regulating intestinal endocrine function, promoting neurotransmitter release, modulating drug action and metabolism, eliminating toxins, and producing numerous compounds that affect the host. Extensive debate exists regarding whether abnormal microbial communities associated with diseases are causally related to the diseases (i.e., susceptibility, initiation, or progression) (Davies et al., 2018; Fan and Pedersen, 2021). An important finding is that the abundance of *Bacteroidetes* and *Lachnospiraceae* in the gut of TMJ mice significantly decreased (Ma et al., 2020). In another study, 16S rRNA sequencing of TMJ mice revealed significant changes in *Bacteroidetes*, *TM7*, *Actinobacteria*, *Tenericutes*, *Verrucomicrobia*, *Cyanobacteria*, *Spirochaetes*, and *Elusimicrobia* (Shen et al., 2023). Additionally, arthritis patients displayed an enrichment of genera such as *Anaerostipes*, *Bifidobacterium*, *Brachyspira*, and *Eggerthella*, while healthy controls had higher levels of genera such as *Faecalibacterium*, *Lachnoclostridium*, *Phascolarctobacterium*, and *Paraprevotella* (Jeyaraman et al., 2023). Using IVW estimation, a statistical method that uses the reciprocal of the variance of the results (square of standard error) as a weight for fitting is used to evaluate the causal impact of 211 gut microbiota on TMD. The analysis of the data in this article suggests that the abundance of 12 bacteria were found to have causal relationships with the progression of TMD. Among them, 11 species of bacteria were found to exacerbate TMD, while Peptococcaceae seemed to had a protective effect. In subsequent stability tests, removing SNP loci that influenced the results did not alter the stability of the outcomes. Therefore, the relationship of the above microbial communities on TMD to be stable and reliable. Reverse MR revealed no evidence of causal effect of TMD on the on the identified gut microbiome.

The relationship between gut microbiota and TMD may be based on the susceptibility to TMD caused by dysbiosis of gut microbiota, or the microbiota and its metabolites acting as promoting factors for TMD. Increasing evidence suggested an association between the family *Lachnospiraceae* and the genus *Ruminococcus* with inflammatory diseases. *Ruminococcus* is a Gram-positive anaerobic bacterium belonging to the phylum *Firmicutes*, and it is one of the 57 species present in 90% of individuals at a median abundance of around 0.1% (Aguilera et al., 2012; Crost et al., 2023). An increase in the abundance of *Ruminococcus* has been observed in patients with ankylosing spondylitis (Vereecke and Elewaut, 2017). Additionally, a correlation exists between the prevalence of musculoskeletal pain (MSKP) in elderly community members and *Ruminococcus* (Shmagel et al., 2018).

Costello et al. observed alterations in the composition of gut microbiota from patients with ankylosing spondylitis (AS), where the increased abundance of bacteria from the *Lachnospiraceae*, *Ruminococceae*, *Rikenellaceae*, *Porphyromonadae*, and *Bacteroideae* appeared at AS (Costello et al., 2015). This study similarly suggested that *Lachnospiraceae ucg-010* and the *Ruminococcus* may play a promoting role in TMD.

In this study, *Peptococcaceae* may have a mitigating or protective effect on the occurrence of TMD. This effect may be associated with their impact on metabolic products. Wen et al. confirmed the correlation of *Peptococcaceae* with various metabolic pathways, including tryptophan and tyrosine, derived from metabolomic and 16S rRNA gene sequencing analyses (Wen et al., 2019).

The analysis of the data in this article also provides some new insights into the effects of drugs in TMD treatment. TMD treatment targets both pain relief and functional improvement. The reports described that a large number of TMD patients had used medication, including anti-inflammatory drugs, over-the-counter painkillers, and even antidepressants, anti-anxiety drugs, and muscle relaxants (Gauer and Semidey, 2015). Although the standard of drug treatment for TMD still lacks evidence-based support, nonsteroidal anti-inflammatory drugs are considered the first choice, and other drugs mentioned above have also been used in combination (Ta and Dionne, 2004). The differences in the abundance of intestinal microorganisms among different populations may lead to different responses to TMD drug treatment. *Lachnospiracea*, a diverse obligate anaerobic bacteria, is abundant in the human gut. In rats administered with low-dose aspirin, alterations in the *Lachnospiracea* family and its interactions with the *Ruminococcaceae* family were observed (Chi et al., 2021). When using medication to treat TMD, changes in the aforementioned bacteria may be potential evaluation indication. Some microbial communities may synergize with malocclusion. Malocclusion is an important cause of TMD, resulting from multiple factors such as genes, occlusal training, and nutritional factors that affect growth and development (Masucci et al., 2020). The synthesis of butyrate by human gut microbiota has significantly improve skeletal muscle mass (Lv et al., 2021). Skeletal muscles, especially chewing muscles and some head and neck muscle groups, have a promoting or inhibiting effect on the development of maxillofacial bones, which may lead to excessive or insufficient development of the jawbone, further leading to malocclusion (Rot et al., 2014). *Peptococcaceae* has been demonstrated to enhance muscle strength through the synthesis of butyric acid, and occlusal muscle strength is an important factor affecting maxillofacial development (Joshi et al., 2014; Parada et al., 2019; Leszczyszyn et al., 2021). The increase in the abundance of *Turicibacter* may reduce the content of bile acids such as lithocholic acid, which in turn leads to a decrease in the intestinal absorption of vitamin D (Guida et al., 2020; Hao et al., 2022). Skeletal development is another factor that affects malocclusion. This article suggests that malocclusion played a partial mediating role in the effects of *Turcicactor*, *Victivalis*, and *Peptococcaceae* on TMD. Regulating the gut microbiota is expected to become a part of the precise therapy of TMD, relying on reducing the inflammatory response around the joints, and providing a suitable microenvironment for the regeneration of surrounding chondrocytes.

This study focuses on exploring the causal relationship between gut microbiota and TMD without delving into the specific regulatory mechanisms. Given the complex interplay among gut microbiota and the broad spectrum of pathogenic factors associated with TMD, there may be intricate cross-interactions among these factors. This underscores the necessity for further comprehensive research to achieve precision in treating TMD. Additionally, the study relies on GWAS data grouped based on epidemiological surveys rather than specific TMD pathology group, which hinders further research on the pathogenic factors of different pathological states of TMD. Therefore, the interpretation of the result still needs to be cautious. Moreover, given that the majority of participant are European descent, extending these research conclusions to other populations requires broader data support.

## 5 Conclusions

In conclusion, this study represents a view in understanding the connections between temporomandibular joint disorders and gut microbiota. These identified microbial signatures associated with TMD contribute to reveal the mechanistic and explore novel therapeutic avenues for individuals precise therapy.

## Data Availability

Publicly available datasets were analyzed in this study. This data can be found here: https://mibiogen.gcc.rug.nl/; https://gwas.mrcieu.ac.uk/datasets/finn-b-TEMPOROMANDIB/.
