# Supplementary material for: Exploring the gut microbiota’s effect on temporomandibular joint disorder: a two−sample Mendelian randomization analysis
Source: Front Cell Infect Microbiol. 2024 Aug 12;14:1361373. doi: 10.3389/fcimb.2024.1361373 (PMC11345233; doi:10.3389/fcimb.2024.1361373)
Supplement: Supplementary file 1 [file Datasheet1.docx]

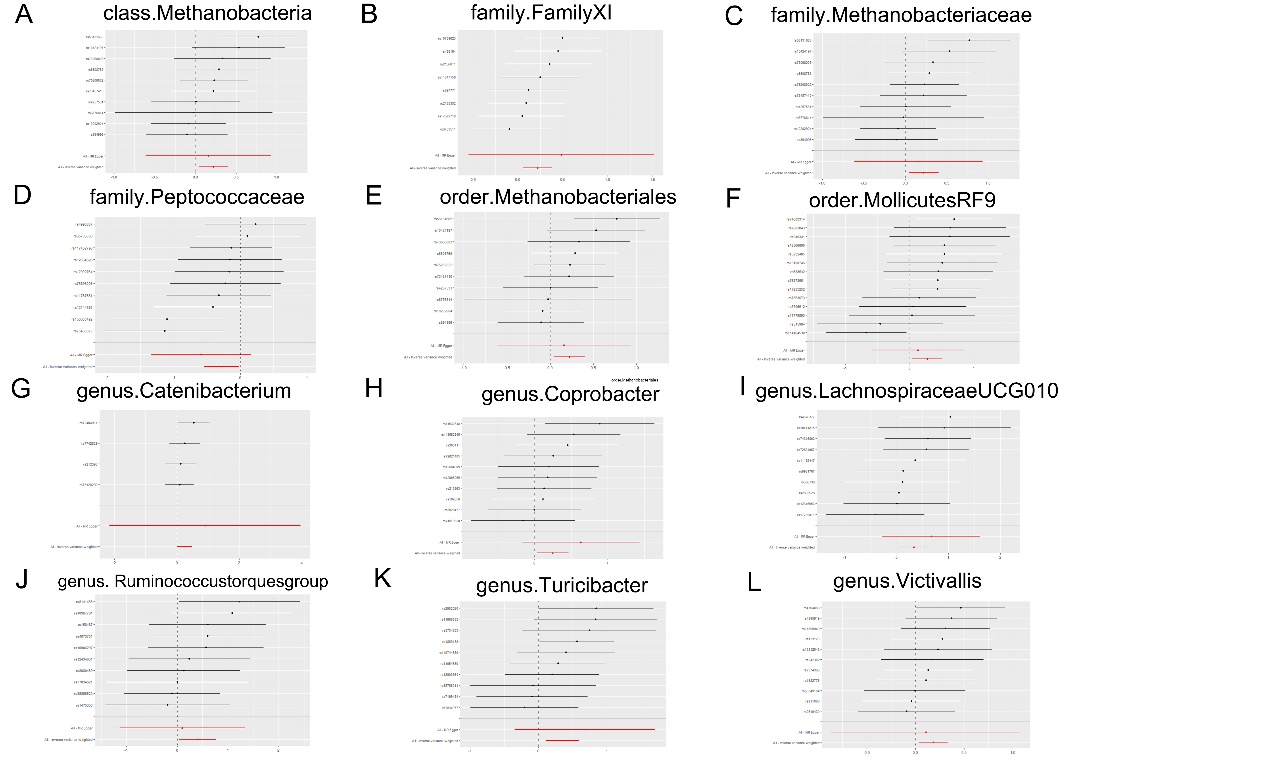


**Supplementary figure 1.** MR forest plot of sensitivity analysis. (A) Forest plot sensitivity analysis of the effect of *class.Methanobacteria* on TMD; (B) Forest plot sensitivity analysis of the effect of *family.FamilyXI* on TMD; (C) Forest plot sensitivity analysis of the effect of *family.Methanobacteriaceae* on TMD; (D) Forest plot sensitivity analysis of the effect of *family.Peptococcaceae* on TMD; (E) Forest plot sensitivity analysis of the effect of *order.Methanobacteriales* on TMD; (F) Forest plot sensitivity analysis of the effect of *order.MollicutesRF9* on TMD; (G) Forest plot sensitivity analysis of the effect of *genus.Catenibacterium* on TMD; (H) Forest plot sensitivity analysis of the effect of *genus.Coprobacter* on TMD; (I) Forest plot sensitivity analysis of the effect of *genus.Lachnospiraceaeucg010* on TMD; (J) Forest plot sensitivity analysis of the effect of *genus.Ruminococcustorquesgroup* on TMD; (K) Forest plot sensitivity analysis of the effect of *genus.Turicibacter* on TMD; (L) Forest plot sensitivity analysis of the effect of *genus.Victivallis* on TMD. TMD: Temporomandibular joint disorder ; MR, Mendelian randomization.


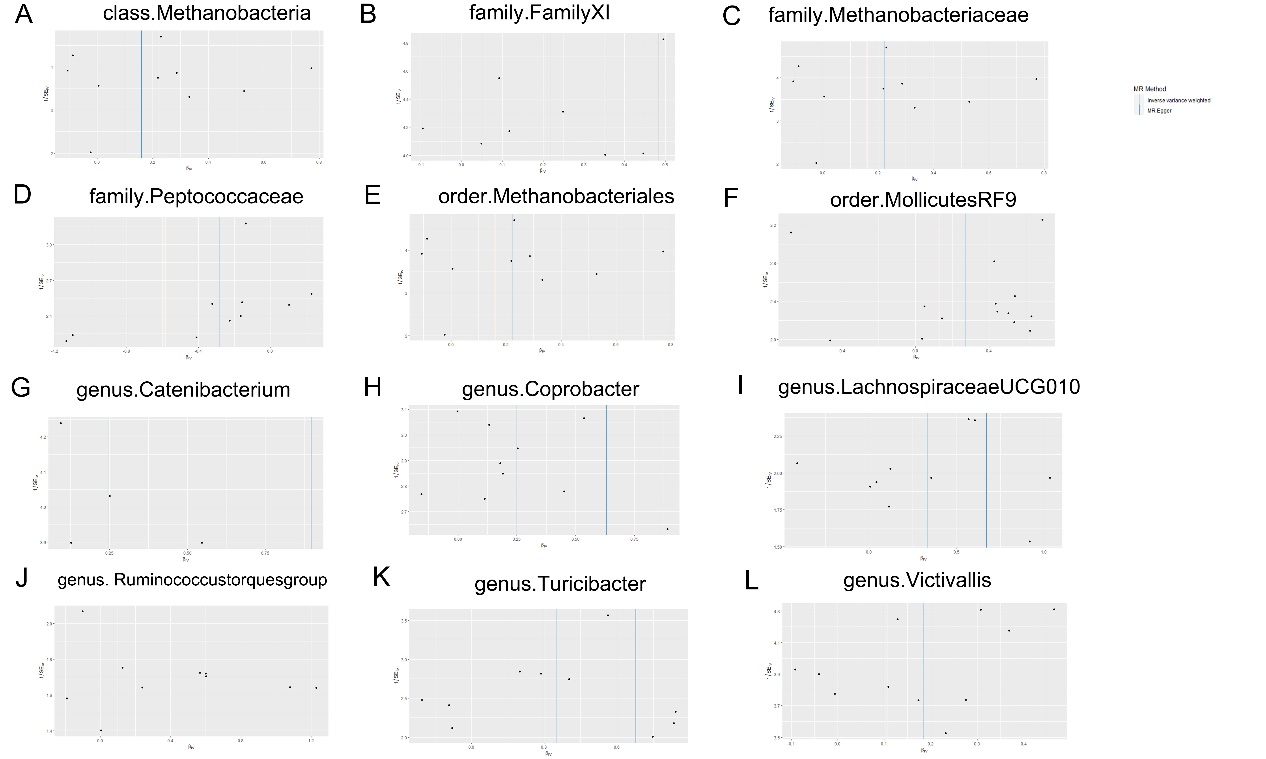


**Supplementary figure 2**. MR funnel plot of sensitivity analysis. (A) Funnel plot sensitivity analysis of the effect of *class.Methanobacteria* on TMD; (B) Funnel plot sensitivity analysis of the effect of *family.FamilyXI* on TMD; (C) Funnel plot sensitivity analysis of the effect of *family.Methanobacteriaceae* on TMD; (D) Funnel plot sensitivity analysis of the effect of *family.Peptococcaceae* on TMD; (E) Funnel plot sensitivity analysis of the effect of *order.Methanobacteriales* on TMD; (F) Funnel plot sensitivity analysis of the effect of *order.MollicutesRF9* on TMD; (G) Funnel plot sensitivity analysis of the effect of *genus.Catenibacterium* on TMD; (H) Funnel plot sensitivity analysis of the effect of *genus.Coprobacter* on TMD; (I) Funnel plot sensitivity analysis of the effect of *genus.Lachnospiraceaeucg010* on TMD; (J) Funnel plot sensitivity analysis of the effect of *genus.Ruminococcustorquesgroup* on TMD; (K) Funnel plot sensitivity analysis of the effect of *genus.Turicibacter* on TMD; (L) Funnel plot sensitivity analysis of the effect of *genus.Victivallis* on TMD. TMD: Temporomandibular joint disorder ; MR, Mendelian randomization.


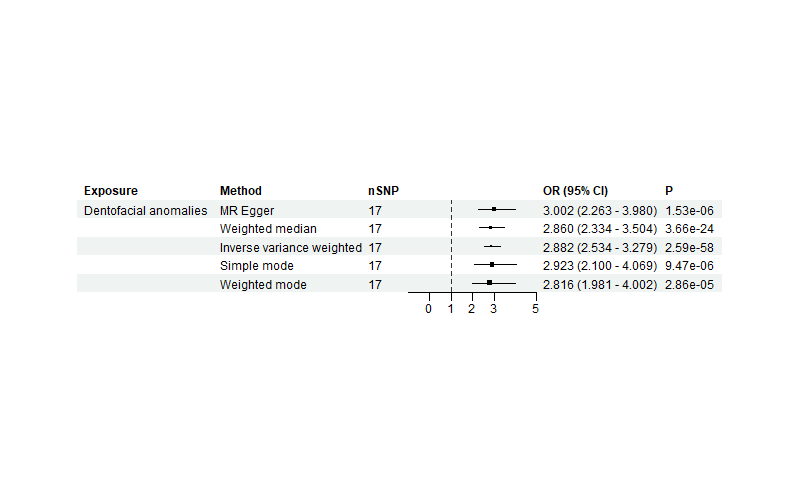


**Supplementary figure 3.** Forest plots for dentofacial anomalies and TMD . OR, odds ratio; CI, confidence interval. *P* < 0.05.


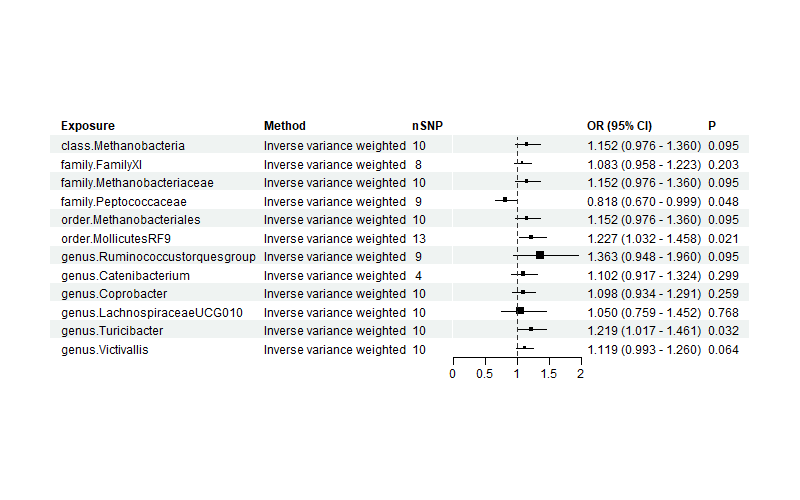


**Supplementary figure 4.** Forest plots for gut microbiome and dentofacial anomalies . OR, odds ratio; CI, confidence interval. *P* < 0.05.


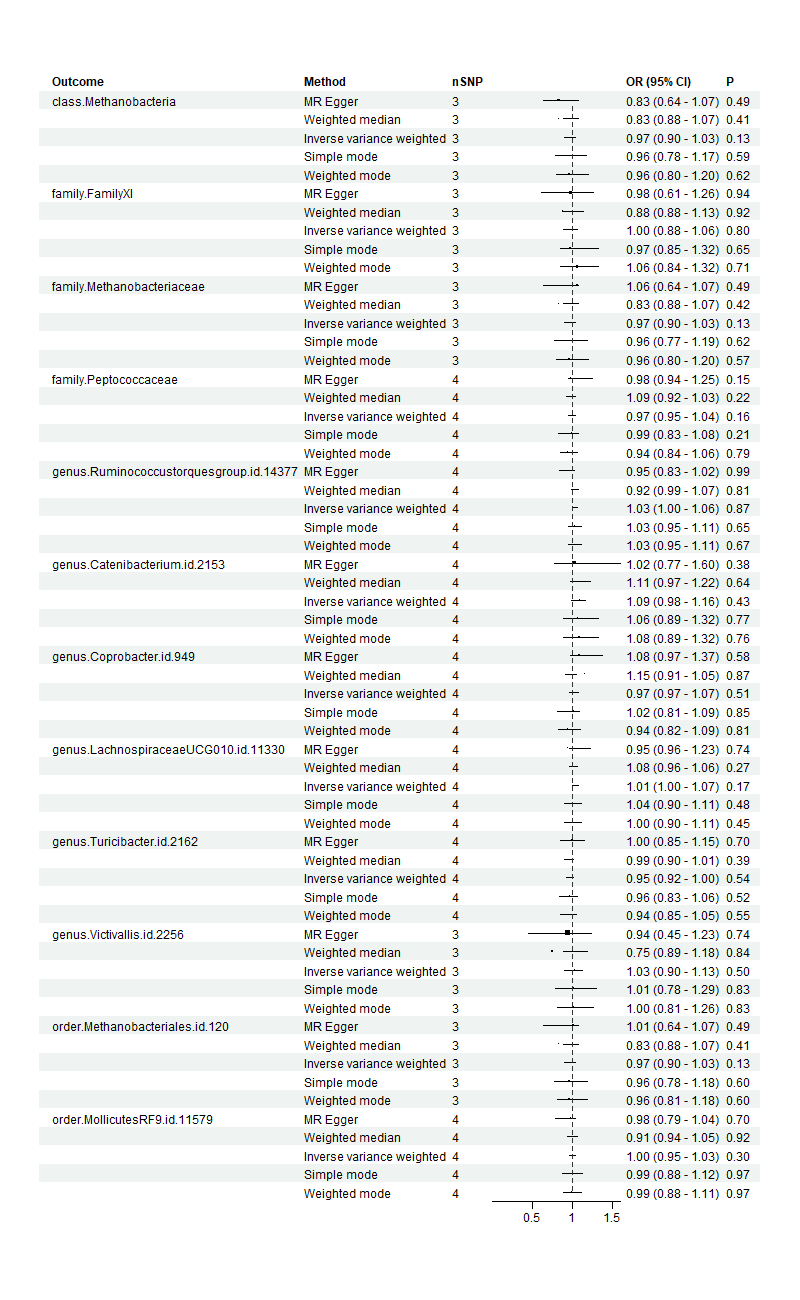


**Supplementary figure 5.** Forest plots for revers gut microbiome and TMD. OR, odds ratio; CI, confidence interval. *P* < 0.05.
